# Supplementary material for: A multicaloric material as a link between electrocaloric and magnetocaloric refrigeration
Source: Sci Rep. 2016 May 25;6:26629. doi: 10.1038/srep26629 (PMC4879550; doi:10.1038/srep26629)
Supplement: Supplementary Information [file srep26629-s1.pdf]

A multicaloric material as a link between electrocaloric and magnetocaloric refrigeration

Hana Ursic[1]*, Vid Bobnar[1], Barbara Malic[1], Cene Filipic[1], Marko Vrabelj[1], Silvo Drnovsek[1], Younghun Jo[2], Magdalena Wencka[3], Zdravko Kutnjak[1]

[1]Jožef Stefan Institute, Jamova cesta 39, 1000 Ljubljana, Slovenia

[2]Spin Engineering Physics Team, Korea Basic Science Institute, Daejeon 34133, Korea

[3]Institute of Molecular Physics, Polish Academy of Sciences, ul. Smoluchowskiego 17, 61-179 Poznań, Poland

*Contact author: hana.ursic@ijs.si (Hana Uršič)

(+386 51 305 154)

Jozef Stefan Institute, Jamova Cesta 39

Ljubljana, Slovenia, 1000

For the microstructural investigation, the PFN-PMW samples were mounted in epoxy, then ground and polished using standard metallographic techniques. The microstructure was examined with a field-emission scanning electron microscope (FE-SEM, JSM-7600F JEOL Ltd., Tokyo, Japan) at 10 kV (fracture-surface and polished surface) and 15 kV (thermally etched surface). The microstructure of the PFN-PMW is relatively dense, uniform, and consists of micron-sized grains, see Figure S1. No secondary phases could be detected.

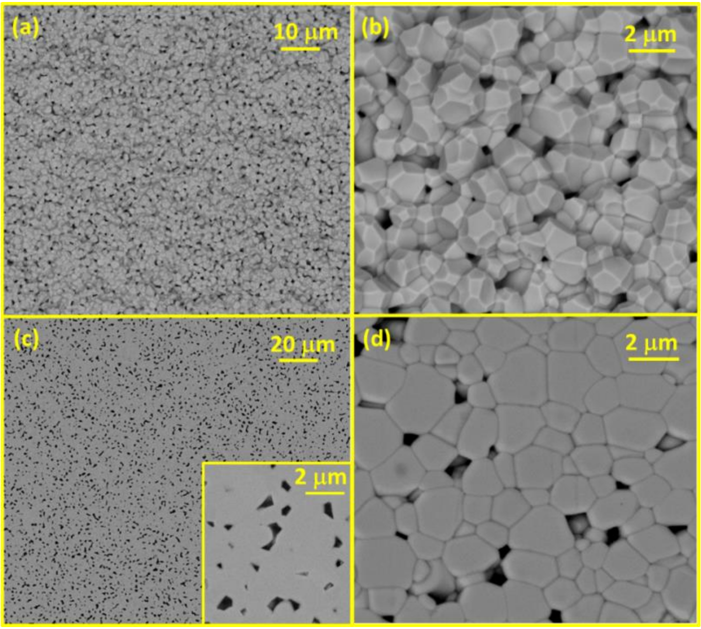

Figure S1: FE-SEM micrographs of the (a) and (b) fracture-surface, (c) polished surface and (d) thermally etched surface of the PFN-PMW sample.

The XRD pattern of the PFN-PMW was recorded with a PANalytical X'Pert PRO MPD (PANalytical, Almelo, Netherlands) diffractometer with Cu–$K_{\alpha1}$ radiation (λ = 1.54056 Å) in the 2θ-range from 10° to 70° using a detector with a capture angle of 2.122°. The exposure time for each step was 100 s and the interval between the obtained data points was 0.034°. The XRD pattern revealing reflections of the perovskite phase is shown in Figure S2. No secondary phases were detected, in agreement with the microstructural analysis.

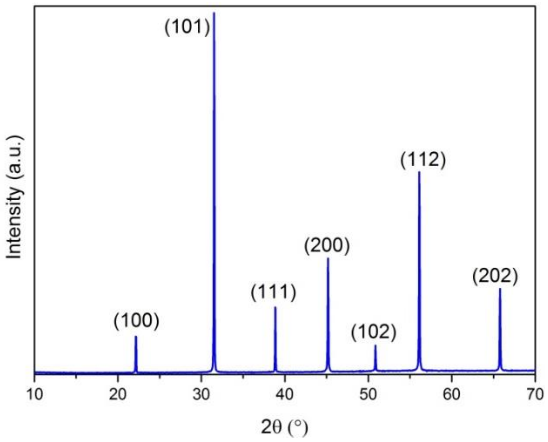

Figure S2: Room-temperature XRD pattern of the PFN-PMW sample. The indexed peaks of the perovskite phase are shown in brackets (ICSD 043788).

The complex dielectric constant $\varepsilon^*(\nu, T) = \varepsilon' - i\varepsilon''$ was measured using an Agilent E4980A Precision LCR meter in the temperature range from 150 K to 310 K at frequencies from 3 Hz to 30 kHz. The amplitude of the probing ac electric signal was 1 V. The temperature was stabilized to within ±0.01 K using a lock-in bridge technique with a Pt100 resistor as the thermometer. Figure S3 shows a broad relaxor dispersive maximum in both the real and imaginary parts of the complex dielectric constant with the ε' peak at ~270 K (for 10 kHz). The polarization-electric field (*P*-*E*) response was measured on a 200-μm-thick sample at a frequency of 100 Hz using a modified Sawyer-Tower bridge. The result, detected at the applied electric field of 18 kV/cm and at 200 K – a typical ferroelectric hysteresis loop – is shown in Figure S4.

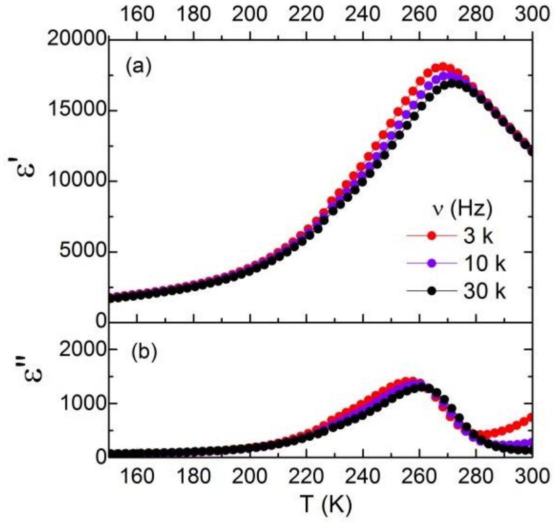

Figure S3: Temperature dependence of the (a) real and (b) imaginary parts of ε^* for the polycrystalline PFN-PMW.

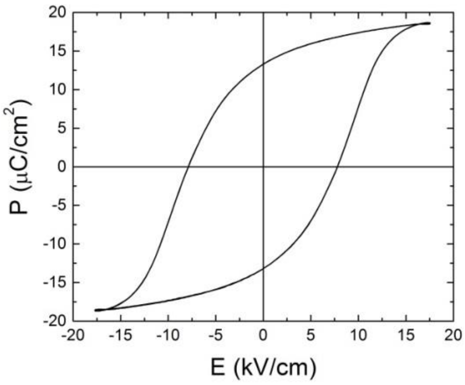

Figure S4: Polarization-electric field (*P*-*E*) response measured at 200 K.

The magnetocaloric and electrocaloric temperature changes were calculated using equations [ref. S1, S2]:

$$(1) \quad \Delta T_{MC} = -\frac{\mu_0\, T}{Cp}\int_{H_1}^{H_2}\left(\frac{dM}{dT}\right)_H dH,$$

$$(2) \quad \Delta T_{EC} = -\frac{T}{\rho\, Cp}\int_{E_1}^{E_2}\left(\frac{dP}{dT}\right)_E dE,$$

where μ_0 is the magnetic permeability of free space ($4\pi \cdot 10^{-7}\,\frac{Vs}{Am}$), ρ is the density and Cp is the specific heat capacity of the material. The Cp was measured using a Physical Property

Measurement System. Figure S5 shows the temperature dependence of *Cp*, which was used in the calculations.

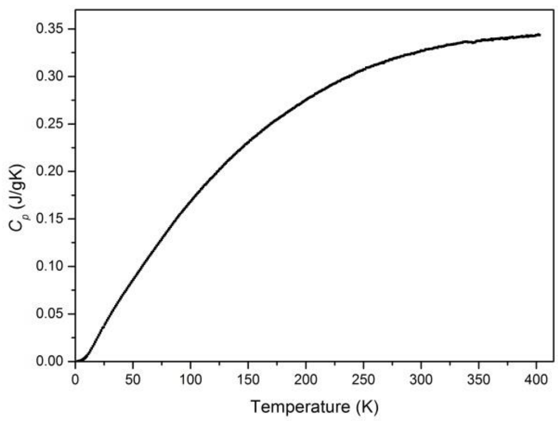

Figure S5: Temperature dependence of the *Cp* for polycrystalline PFN-PMW.

Literature

[S1] A. Kitanovski, J. Tušek, U. Tomc, U. Plaznik, M. Ožbolt, A. Poredoš, *Magnetocaloric energy convertion*, ISSN 1865-3529, Springer International Publishing Switzerland, Switzerland, 2015.

[S2] Z. Kutnjak, B. Rožič, R. Pirc, *Electrocaloric effect: theory, measurements, and applications*, Wiley Encyclopedia Of Electrical And Electronics Engineering, John Wiley & Sons, Inc., New Jersey, 2015.
